# Glutamate spillover drives robust all-or-none dendritic plateau potentials—an *in silico* investigation using models of striatal projection neurons

**DOI:** 10.3389/fncel.2023.1196182

**Published:** 2023-06-29

**Authors:** Daniel Trpevski, Zahra Khodadadi, Ilaria Carannante, Jeanette Hellgren Kotaleski

**Affiliations:** ^1^Science for Life Laboratory, Department of Computer Science, KTH Royal Institute of Technology, Stockholm, Sweden; ^2^Department of Neuroscience, Karolinska Institutet, Stockholm, Sweden

**Keywords:** glutamate spillover, plateau potentials, NMDA spikes, gating function, computational modeling, nonlinear dendritic computation, clustered synapses, magnesium block of NMDA receptors

## Abstract

Plateau potentials are a critical feature of neuronal excitability, but their all-or-none behavior is not easily captured in modeling. In this study, we investigated models of plateau potentials in multi-compartment neuron models and found that including glutamate spillover provides robust all-or-none behavior. This result arises due to the prolonged duration of extrasynaptic glutamate. When glutamate spillover is not included, the all-or-none behavior is very sensitive to the steepness of the Mg^2+^ block. These results suggest a potentially significant role of glutamate spillover in plateau potential generation, providing a mechanism for robust all-or-none behavior across a wide range of slopes of the Mg^2+^ block curve. We also illustrate the importance of the all-or-none plateau potential behavior for nonlinear computation with regard to the nonlinear feature binding problem.

## 1. Introduction

Plateau potentials are all-or-none voltage elevations that occur in neuronal dendrites in various brain regions, including striatal projection neurons (SPNs), which last tens to hundreds of milliseconds (Plotkin et al., 2011; Oikonomou et al., 2014; Du et al., 2017). They are involved in processing sensory information, as well as triggering synaptic plasticity (Lavzin et al., 2012; Xu et al., 2012; Gambino et al., 2014; Kumar et al., 2018). Clustered synapses, which facilitate the induction of dendritic NMDA spikes and plateau potentials, have been shown to form during development and learning (see e.g., review by Kastellakis and Poirazi, 2019). Plateau potentials are generated on stretches of dendrites around and above 20 μm of length, where the N-methyl-D-aspartate (NMDA) receptors that mediate them must have a cumulative conductance above a threshold level (Antic et al., 2010). Experimentally, plateau potentials have been evoked by activating clusters of synapses by glutamate uncaging or by stimulating stretches of dendrites by glutamate iontophoresis or repeated synaptic stimulation (Milojkovic et al., 2004; Major et al., 2008; Plotkin et al., 2011; Oikonomou et al., 2012; Du et al., 2017; Kumar et al., 2018; Gao et al., 2021). In the cases of progressively increasing glutamate stimulus in equal increments, a supralinear, all-or-none response in the amplitude of the somatic voltage is observed (Major et al., 2008; Oikonomou et al., 2012; Gao et al., 2021). In this study, we deal with the question of how to model the all-or-none behavior of plateau potentials in multicompartment neuron models and discuss the implications of the model for the precise mechanism of generation of plateau potentials experimentally and *in vivo*.

**Figure 1:**
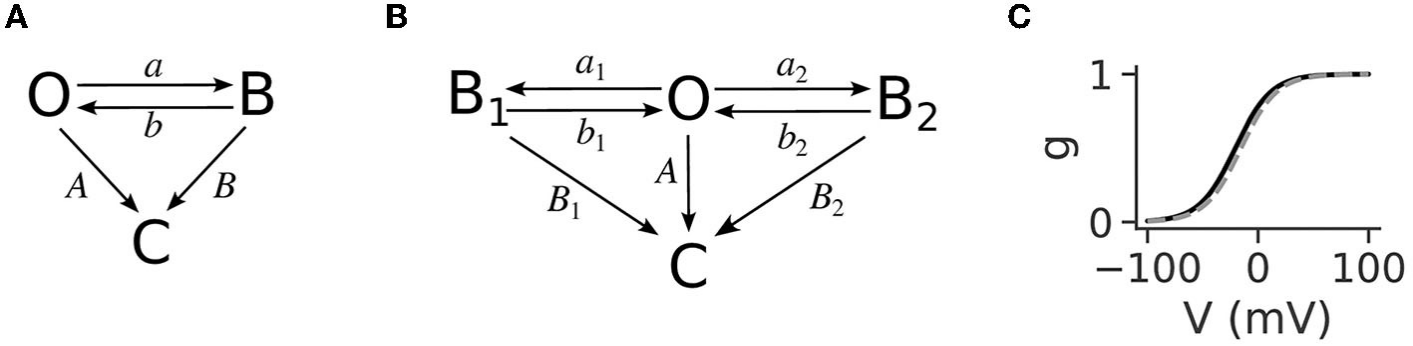
**(A)** The 3-state model for the functioning of open NMDARs, reproduced from Jahr and Stevens (1990b). It has three states: open (O), blocked (B), and closed (C). The transition rates between the open and blocked states, *a* and *b*, are voltage-dependent, while the transitions to the closed state, *A* and *B*, are not. The rate of blocking the open state, a, is additionally linearly dependent on [Mg^2+^]. Transmitter removal causes the transitions to the closed state. **(B)** The four-state model for the functioning of NMDARs used in Jahr and Stevens (1990b) to derive the gating function for the Mg^2+^ block. It contains an additional blocked state, B_1_, the transition to which (a_1_) is [Mg^2+^]-independent. The other transition rates are analogous to the three-state model, and are given in Table 1. **(C)** The gating function is expressed as a sigmoidal curve and shows the proportion of open NMDARs as a function of voltage. The steepness of the curve and its position along the x-axis, described with the parameters α and η in Equation (4) are usually fitted from macroscopic NMDA current measurements. The black line is the curve with the parameter values determined in Jahr and Stevens (1990b), and the gray dashed line is the curve with the corrected parameter value for η as in Ecker et al. (2020), which accounts for the junction potential.

The NMDA receptors are ionotropic receptors activated by glutamate and glycine as a co-factor that are additionally blocked by Mg^2+^ ions at resting membrane potentials. The amount of blockage depends on the local voltage, and it has been determined that this dependence is a sigmoidal nonlinearity (Figure 1C), where higher voltage relieves the block (Jahr and Stevens, 1990b). The generation mechanism is a positive feedback loop that starts by partially alleviating the Mg^2+^ block on NMDA receptors (NMDARs) when the dendritic voltage reaches a threshold value, usually around −50 mV. This causes the flow of an inward current through the NMDA receptors, raising the voltage in a cycle that progressively alleviates more of the Mg^2+^ block and allows for more inward current. Thus, a sustained voltage elevation is produced, which lasts as long as there is glutamate to bind to the NMDARs. The sigmoidal shape of the voltage nonlinearity is a crucial ingredient for the all-or-none behavior of the plateau potentials since, for linear increases in voltage, it provides a “sudden” relief of Mg^2+^ ions from the NMDARs. The binding of neurotransmitters to the NMDAR initiates a transition from the closed receptor state to the open state. The behavior of an NMDAR in the open state is described with the diagram in Figure 1A, also called the three-state model, since it includes three receptor states: open, blocked, and closed. An NMDAR in the open state can transition to two different non-conducting states, closed and blocked. The transitions between the open and blocked states depend on Mg^2+^ concentration (denoted as [Mg^2+^]) and voltage, while the transitions from the open and blocked states to the closed state are voltage- and [Mg^2+^]-independent. Voltage crossing the −50 mV threshold unblocks some of the receptors, initiating the self-sustaining positive feedback loop. Neurotransmitter removal closes the receptor pore (the receptor transitions to the closed state), thus stopping the inward current and lowering the voltage. Lowering the voltage causes remaining open receptors (with bound neurotransmitter) to transition to the blocked state, which because of the sigmoidal nonlinearity rapidly lowers the voltage back to the resting state.

**Table 1:** Transition rates for the four-state model reproduced from Jahr and Stevens (1990a,b).

**Transition rate**	**Value**
*a*_1_	exp(−0.016·*V*−2.91) [ms^−1^]
*a*_2_	C · exp(−0.045·*V*−6.97) [μM^−1^ ms^−1^]
*b*_1_	exp(0.009·*V*+1.22) [ms^−1^]
*b*_2_	exp(0.017·*V*+0.96) [ms^−1^]
*A*	exp(−2.847) [ms^−1^]
*B*_1_	exp(−0.693) [ms^−1^]
*B*_2_	exp(−3.101) [ms^−1^]

The transition rates were derived by single-channel analysis. *C* represents the magnesium concentration in μM and *V* the voltage in mV.

### 1.1. NMDAR state diagrams and the gating function

The three-state model has been shown to be suitable for high Mg^2+^ concentrations ([Mg^2+^]>0.2 mM), but it can be easily expanded to take into account lower concentrations by introducing an additional, [Mg^2+^]-independent, blocked state (Figure 1B, the four-state model; Jahr and Stevens, 1990a). All the transition rates of the four-state model have been derived from single-channel measurements and reported in Table 1 (reproduced from Jahr and Stevens, 1990a). The rate of leaving the open state in the three-state model (*O*→*B*) is *a*, and the corresponding rate for the four-state model (*B*_1_←*O*→*B*_2_) is *a*_1_+*a*_2_. The transition rate from blocked to open is *b* in the three-state model, whereas the corresponding rate in the four-state model is *b*_1_*a*_1_/(*a*_1_+*a*_2_)+*b*_2_*a*_2_/(*a*_1_+*a*_2_) because the rate of leaving each blocked state is weighted by the probability that particular state had been entered. Similarly, the closing rate from the blocked state is *B* for the three-state model and *B*_1_*a*_1_/(*a*_1_+*a*_2_)+*B*_2_*a*_2_/(*a*_1_+*a*_2_). The Mg^2+^ block is expressed with a gating function, *g*(*V*), which gives the fraction of open NMDARs for a given voltage value. As shown in Jahr and Stevens (1990b) it is possible to express *g*(*V*) in terms of the transition rates as:


(1) $$\begin{array}{l} g\left(V\right)\;=\;\frac{1}{1+\frac{\left(a_{1}+a_{2}\right)\left(a_{1}B_{1}+a_{2}B_{2}\right)}{Aa_{1}\left(b_{1}+B_{1}\right)+Aa_{2}\left(b_{2}+B_{2}\right)}}. \end{array}$$

The single-channel analysis showed that *b*_1_ and *b*_2_ are much larger than *B*_1_ and *B*_2_ (also visible in Table 1); hence, the following is a good approximation:


(2) $$\begin{array}{l} g\left(V\right)\;\approx \;\frac{1}{1+\frac{\left(a_{1}+a_{2}\right)\left(a_{1}B_{1}+a_{2}B_{2}\right)}{Aa_{1}b_{1}+Aa_{2}b_{2}}}. \end{array}$$

Moreover, when [Mg^2+^] is greater than a few hundred micromolar, *a*_2_ becomes much larger than *a*_1_ since the former increases linearly with [Mg^2+^] while the latter is independent of it, and hence *g*(*V*) for physiological [Mg^2+^] can be further approximated as:


(3) $$\begin{array}{l} g\left(V\right)\;\approx \;\frac{1}{1+\frac{B_{2}a_{2}}{Ab_{2}}}\approx \frac{1}{1+\frac{Ba}{Ab}}. \end{array}$$

Finally, taking into account the transition rates in Table 1, the gating function can be written in its most commonly used form:


(4) $$\begin{array}{l} g\left(V\right)\;=\;\frac{1}{1+\eta \;\left[{\text{Mg}}^{2+}\right]e^{-\alpha V}}, \end{array}$$

where α is the steepness of the curve, η is related to the voltage value *V*_1/2_ where half of the receptors are free of Mg^2+^ according to $V_{1/2}=\frac{\text{ln}\left(\eta \left[{\text{Mg}}^{2+}\right]\right)}{\alpha }$ , [Mg^2+^] is the concentration of extracellular Mg^2+^ in mM and V is the membrane potential in mV (see Supplementary material for details and Supplementary Figure 1). As described in the Supplementary material, the values of α and η are determined by the transition rates between different states in the 4-state model, which in turn are determined by the structural properties of the NMDARs.

The gating function adequately describes the behavior not only of single channels but also of macroscopic NMDAR currents, which allows for the determination of the parameters α and η from macroscopic current measurements. Such macroscopic current measurements from NMDARs, as well as gating functions fitted to such measurements, have been reported numerous times since the work of Jahr and Stevens (1990b), and are summarized in Table 2. For completeness, in Table 2 we have added parameter values which were not estimated in the original articles. The fitting procedure, as well as the results of the fittings, are described in the Methods and Results sections, respectively. Table 2 shows a natural variability in the parameters of the Mg^2+^ block gating function which depends on the NMDAR subunit composition, as well as on the concentration of other intra- and extra-cellular ions (Qian et al., 2005; McMenimen et al., 2006; Qian and Johnson, 2006; Retchless et al., 2012). Since the sigmoidal Mg^2+^ block is a crucial ingredient for the all-or-none behavior of plateau potentials, we investigate the effect of the parameter variability on the behavior of the plateau potentials.

**Table 2:** Gating function parameter values from experimental data.

	**NMDAR type**	**[Mg] (mM)**	**α (/mV)**	**η (/mM)**	**Cell type**	**Ionic concentration (mM)**
Nowak et al. (1984)	?	0.5	0.04	1.33	Mouse mesencephalic or striatal neurons	140 [Na]_o_
Jahr and Stevens (1990b)	?	1	0.062	0.28	Rat hippocampal neurons	165 [Na]_o_ / 150 [Cs]_i_
Chen and Huang (1992)	?	0.03	0.05	0.49	Trigeminal subnucleus caudalis neurons	140 [Na]_o_ / 125[Cs]_i_
Sharma and Stevens (1996)	N1/2A	3	0.06	0.28	HEK293 cells	145 [Na]_o_ / 160[Cs]_i_
McMenimen et al. (2006)	N1/2B	2	0.06	0.42	Xenopus laevis oocytes	96 [Na]_o_
	N1/2B	0.2	0.05	3.3		96 [Na]_o_
Chiu and Carter (2022)	?	1	0.074	0.11	CD1 mice
Layer 2/3 pyramidal neurons	155 [Na]_o_, 2 [Ca]_o_, 2.5 [K]_o_ / 130 [Cs]_i_ or 128 [K]_i_
	?	0.7	0.074	0.104		155 [Na]_o_, 1.2 [Ca]_o_, 4.2 [K]_o_ / 130 [Cs]_i_ or 128 [K]_i_
	?	0.8	0.071	0.119		155 [Na]_o_, 1.3 [Ca]_o_, 3.8 [K]_o_ / 130 [Cs]_i_ or 128 [K]_i_

Parameter values are determined from fits to examples of experimental data. Fits for all articles except Jahr and Stevens (1990b) were done as described in Section 2.

### 1.2. Computational models of dendritic plateau potentials

Studies that model plateau potentials and/or NMDA spikes fall into two groups. In the first group are studies that simply include the voltage-dependent Mg^2+^ block of the NMDARs in an NMDA synapse model and activate a cluster of such synapses on a dendrite to generate the plateau potential. The second group comprises one recent study that includes the effect of glutamate spillover when modeling plateau potentials (Gao et al., 2021). Glutamate spillover is thought to occur when a cluster of synapses is stimulated more strongly or repeatedly, and the amounts of exocytosed glutamate surpass the ability of astrocytes to take it up, thus causing it to spill over from the synaptic cleft into the space surrounding the spine necks and dendritic shafts. Here it stimulates extrasynaptic NMDARs (eNMDARs), which can aid the generation of plateau potentials (Chalifoux and Carter, 2011). The eNMDARs have also been found to have a substantial contribution to neuronal up-states in striatal SPNs, which in turn have been hypothesized to be driven by dendritic plateau potentials (Oikonomou et al., 2014; Garcia-Munoz et al., 2015).

None of the studies have explicitly focused on modeling the all-or-none behavior of the plateau potentials. However, all studies have changed the originally reported Mg^2+^ block parameters by Jahr and Stevens (1990b) without explicitly stating a reason for the change. The parameter values for α, η, and [Mg^2+^] that have been used in the different studies are exemplified in Table 3. In addition, all of these studies except one use steeper sigmoid curves compared to the one reported by Jahr and Stevens (1990b).

**Table 3:** Examples of studies that have modeled plateau potentials and the parameters used for the Mg^2+^ block.

	**[Mg] (mM)**	**α (/mV)**	**η (/mM)**
Rhodes (2006)	1 and 2	0.08	0.28
Major et al. (2008)	1.8	0.08	0.11
Farinella et al. (2014)	1	0.08	0.3
Poleg-Polsky (2015)	1	0.08	0.25
Doron et al. (2017)	1	0.08	0.28 and 1.45
Du et al. (2017)	1	0.07	0.33
Dorman et al. (2018)	1.4	0.099	0.055
Kumar et al. (2018)	1	0.08	0.25
Ecker et al. (2020)	1	0.062	0.38
Gao et al. (2021)	1	0.08	0.25

Ecker et al. (2020) have corrected η for the omitted junction potential in Jahr and Stevens (1990b), and use the same value for α. Note that Major et al. (2008) have used a different form of the equation, and we have converted their parameter values to match the form in Equation (4).

We analyze the two different types of models of generating plateau potentials and find that the all-or-none behavior is very sensitive to the steepness of the Mg^2+^ block when no glutamate spillover is included in the model and, conversely, very robust with respect to the steepness of the Mg^2+^ block when glutamate spillover is included. Without glutamate spillover, in order to obtain the all-or-none behavior of the plateau potentials, one needs to increase the steepness of the Mg^2+^ block since the originally reported curve is usually too shallow and provides graded rather than all-or-none voltage elevations. In light of the variability in the steepness of the gating function (Table 2), these results indicate that glutamate spillover might be the reason why plateau potentials exhibit a robust all-or-none quality in experimental recordings.

A robust all-or-none plateau potential behavior can significantly improve nonlinear computation, as we illustrate for the nonlinear feature binding problem. We conclude by discussing the implications of the glutamate spillover model in light of the many NMDAR isoforms, the experimentally observed all-or-none behavior of plateau potentials, as well as the predicted role for dendritic computations.

## 2. Methods

The code implementation is done in Python 3, and the simulations in NEURON (Carnevale and Hines, 2005) are used via the Python interface.

### 2.1. Neuron models

The SPN model is taken from the collection of models published in Lindroos and Hellgren Kotaleski (2021). Spines in the neurons are added as additional compartments consisting of a neck and head with lengths and diameters l_neck_ = 0.5 μm, l_head_ = 0.5 μm, and d_neck_ = 0.125 μm, d_head_ = 0.5 μm, respectively. Axial resistance in all compartments is 150 Ohm · cm, except for the spine neck, where it is 1, 130 Ohm · cm (Dorman et al., 2018). Spines contain the inwardly rectifying potassium channel, with a conductance equal to that of the parent dendritic shaft segment. Voltage-gated calcium channels of types R (Ca_v_2.3), T (Ca_v_3.2 and Ca_v_3.3), and L (Ca_v_1.2 and Ca_v_1.3) are added to the spines as well, and their conductances have been manually tuned to match the relative proportions determined in Carter and Sabatini (2004), and Higley and Sabatini (2010), as well as calcium concentration amplitudes arising from backpropagating action potential (bAP) stimulation as in Figure 2 of Shindou et al. (2011).

### 2.2. Background synaptic noise

Background synaptic noise is implemented by adding synapses across the dendrites according to the excitatory and inhibitory synapse densities reported in Cheng et al. (1997). These synapses are not on spines. We only explicitly model spines where we have clustered synaptic inputs. To increase the speed of the simulations, in compartments where the synaptic density specifies adding more than one synapse, only one synapse was added with scaled-up input frequency to account for the actual number of synapses that would need to arrive in that compartment.

### 2.3. Synaptic inputs

Synaptic inputs consist of AMPA and NMDA synapses located on spines within clusters. Glutamate spillover is modeled by including extrasynaptic NMDA conductances on the dendritic shafts situated directly beneath the spines. For glutamate spillover, we put as many extrasynaptic NMDA synapses as the NMDA synapses on the spines. The AMPA and NMDA synapse models are taken from Gao et al. (2021), which are a variation of the saturating synapse models in Destexhe et al. (1994) implemented in the NEURON simulator (Carnevale and Hines, 2005). These are kinetic models which operate according to two different kinetic schemes depending on the presence of neurotransmitters. When neurotransmitters arrive at the postsynaptic site, receptor dynamics are described by a kinetic scheme that models switching between closed (*C*) and open (*O*) states with rates of α and β:


(5) $$\begin{array}{l} C+T\overset{\alpha }{\underset{\beta }{⇌}}O, \end{array}$$

where *T* is the transmitter concentration. After neurotransmitter has been cleared from the synaptic cleft, receptor dynamics evolves according to a kinetic scheme that describes just the closing of the receptor with a rate β:


(6) $$\begin{array}{l} C\overset{}{\underset{\beta }{\leftarrow }}O \end{array}$$

When a presynaptic spike arrives, neurotransmitter levels are assumed to always reach a fixed saturating concentration, T_max_, in the synaptic cleft, i.e., are represented by a pulse with amplitude T_max_ and duration of T_dur_. A presynaptic spike that arrives while the neurotransmitter pulse is still on lengthens the pulse duration by T_dur_.

Extrasynaptic receptors are modeled by the same kinetic scheme, with two differences: (i) T_dur_ is much longer, modeling the spillover effect of glutamate in the extrasynaptic space, and (ii) the synaptic spike there arrives with a delay of 1.5 ms after the glutamate threshold has been reached (taken from Szapiro and Barbour, 2007, as we describe in the next section). The channel properties of NMDARs and eNMDARs have been reported to be similar (Clark et al., 1997).

We use the model given in Gao et al. (2021) but with modified parameter values, given in Table 4. For example, in the simulation code for the model in Gao et al. (2021) different maximal transmitter concentration, T_max_, for the synaptic AMPA and NMDA synapses is used. Although difficult to justify physiologically, this difference would not affect the results in Gao et al. (2021), since the AMPA synapses are only used to activate the NMDARs and eNMDARs, and since the effect of the different concentrations can be offset with suitable values for the synaptic conductances. We have nevertheless used the same T_max_ for both AMPARs and synaptic NMDARs. In addition, the model in Gao et al. (2021) uses the same T_max_ for NMDARs and eNMDARs. We have changed this, since, even though the extrasynaptic glutamate concentration is not precisely known, extrasynaptic spillover-induced currents are very sensitive to low-affinity AMPA antagonists, suggesting lower extrasynaptic glutamate concentrations (Szapiro and Barbour, 2007). Low glutamate concentrations during spillover have also been reported in Okubo et al. (2010). As for the maximal synaptic glutamate concentration, T_max_, it has been found to be very close to 1 mM at cultured hippocampal synapses (Clements et al., 1992).

**Table 4:** Parameters for the synapse models with glutamate spillover.

	**g_max_ (nS)**	**w**	**T_dur_ (ms)**	**T_max_ (mM)**	**α [/(ms mM)]**	**β (/ms)**	***K*_*d*_ (μM) (nS)**
AMPA	1.5	0.4	1	1	12.5	0.25	20
NMDA	3.5	0.4	1	1	4	0.01	2.5
eNMDA	3.5	0.4	50+200w	0.2	4	0.01	2.5

In the cases without spillover the NMDA conductance in the spines is the same as in the cases with spillover, i.e., NMDA g_max_ = 3.5 nS, and eNMDA g_max_ = 0 nS. The synaptic weight parameter is w (described in the text).

We have used a short T_dur_ for NMDARs (the same as for AMPARs), and long T_dur_ for eNMDARs. Lastly, we have modified the α and β parameters for AMPARs to more realistic values. Also note that we have denoted transmitter duration and transmitter concentration with T_dur_ and T_max_, respectively, which are given with C_dur_ and C_max_ in the simulation code in Gao et al. (2021).

eNMDARs have been found to have both diffuse and punctual distribution along dendritic shafts, and electrophysiological measurements have reported their conductance to be 20–60% of the total NMDAR conductance (Harris and Pettit, 2007; Petralia, 2012; Papouin and Oliet, 2014). Even though synapses are represented as punctual inputs in NEURON, the distribution of eNMDAR synapses along 20–30 μm of a dendrite resulted in us effectively modeling a diffuse distribution of eNMDARs. The maximal conductance of an extrasynaptic NMDA synapse in the model is the same as the maximal conductance for a synaptic NMDA synapse. The total synaptic surface area in the clustered spines is around 10% of the surface area of the dendritic shaft where a cluster is situated, making the density of eNMDARs 10 times smaller than the density of NMDARs on the spine.

### 2.4. Evoking plateau potentials

Plateau potentials are evoked by clustered synaptic input. Clusters ranged from 1 to 40 synapses for the model without spillover and from 1 to 20 synapses for the models with spillover; in SPNs, which are spherically symmetric, a cluster is placed on one of the dendrites, ~120–140 μm from the soma. The difference in the size of the clusters for the cases without and with glutamate spillover is in order to compare the same total NMDAR conductance in both cases. Since both cases have equal spine NMDAR conductance and the case without spillover has no eNMDARs, this requires that it possesses either a twice larger cluster size or a twice larger spine NMDAR conductance to match the total NMDAR conductance in the spillover scenarios; we opted for the former. Clustered synaptic input consists of one presynaptic spike per synapse, all arriving randomly within a 30 ms interval. When no glutamate spillover is modeled, synaptic inputs arrive only to the spines. For glutamate spillover, the same presynaptic spike is delivered to the extrasynaptic NMDA conductance placed in the dendritic shaft under that spine. The study by Gao et al. (2021) models clusters ranging from 10 synapses upwards, which leaves the question of how to treat glutamate spillover for clusters with fewer than 10 synapses open. We have explored two scenarios, which we call thresholded spillover and accumulative spillover. In thresholded spillover we assume glutamate spills over suddenly and simultaneously to all eNMDARs once a glutamate threshold is reached, with a delay of 1.5 ms. The delay is due to experimental data indicating spillover currents arise after 1–2 ms of synaptic currents (Szapiro and Barbour, 2007). In accumulative glutamate spillover there is no threshold for glutamate, and a presynaptic spike delivered to a synaptic NMDA synapse is also delivered to the corresponding extrasynaptic NMDA conductance after a delay of 1.5 ms. This means that every synaptic activation causes spillover and activates the corresponding eNMDARs. An additional difference between the model in Gao et al. (2021) and our study is that in Gao et al. (2021), larger synaptic clusters also have synapses with bigger weights (parameter w) and longer transmitter pulses (parameter T_dur_), whereas in our study we have kept these parameters fixed when increasing cluster size.

The glutamate threshold in thresholded spillover is implemented in the following way. Each NMDA synapse has a weight parameter (w), which is a dimensionless number between 0 and 1 that scales the maximal conductance of the NMDAR. We have set up the model so that a cluster of at least ten synapses with a weight >0.4 needs to be activated to reach the threshold level of glutamate at which spillover occurs, and a spike is delivered to the eNMDARs. We implemented this in NEURON by adding the NMDA synaptic weight to a variable that conceptually models the concentration of glutamate each time a spike arrives at a spine. Added weights are normalized so that ten activated synapses with a weight >0.4 reach the glutamate threshold. To implement this variable, we have in fact used a NEURON integrate-and-fire cell that generates a spike to the eNMDARs in the cluster once it reaches a threshold value of 1.

The two glutamate spillover scenarios we consider are idealized scenarios, and biological glutamate spillover is most likely somewhere in between, varying among brain regions.

### 2.5. Calculation of phase plots for a single membrane compartment

To investigate the role of local membrane conductances in generating plateau potentials, we performed phase plot analysis for a single electrical compartment which we aimed to make similar to a dendritic region where plateau potentials are evoked. For this purpose, in the single compartment, we inserted the ionic channels Na_f_, K_af_, K_as_, K_ir_, K_dr_ with maximal conductances taken from a dendritic segment in the model SPN at ~120 μm from the soma, where clustered inputs are located in most of the results showing voltage traces. (In NEURON nomenclature, the dendritic segment is located at position 0.6 of dendrite 3.) Calcium channels were omitted to simplify the analysis since they have been found to have a minor effect on the voltage, and SK and BK channels have also been omitted since they depend on intracellular calcium concentration, which we do not model in the single compartment. The equation describing the current in the single membrane compartment is:


$$\begin{array}{l} \sum I_{\text{ion}}+I_{\text{NMDA}}+I_{\text{leak}}+I_{c}=0, \end{array}$$

or


$$\begin{array}{l} C_{m}\overset{•}{V}=-\sum I_{\text{ion}}-I_{\text{NMDA}}-I_{\text{leak}}, \end{array}$$

where


$$\begin{array}{lll} I_{\text{ion}} & = & g_{\text{ion}}G_{\infty }^{\text{ion}}\left(V-E_{\text{ion}}\right)\\ I_{\text{NMDA}} & = & g_{\text{max}}^{\text{NMDA}}g\left(V\right)\left(V-E_{\text{NMDA}}\right),\\ I_{\text{leak}} & = & g_{\text{leak}}\left(V-E_{\text{leak}}\right). \end{array}$$

In the equations above $I_{c}=C_{m}\overset{•}{V}$ is the capacitive current, *E*_ion_, *E*_NMDA_, and *E*_leak_ are the reversal potentials and *g*_ion_, $g_{\text{max}}^{\text{NMDA}}$, and *g*_leak_ conductances of the ionic, NMDAR, and leak currents, respectively, and *g*(*V*) is the gating function describing the Mg^2+^ block. The construction of the phase plots consists of calculating the stationary membrane current as dependent on the voltage (as if a voltage value has been “on” for a long time), which means that the gating variables in the ion channels have their asymptotic, time-independent values. These are denoted as $G_{\infty }^{\text{ion}}$. For example, for the Na_f_ channel, this variable is $G_{\infty }^{\text{Naf}}=m_{\infty ,\text{Naf}}^{3}\cdot h_{\infty ,\text{Naf}}$. Since, in addition to the NMDA current, bistable membrane behavior requires a shunting current of longer duration, such as K_ir_ or GABA_B_ currents (comparable to the duration of NMDA currents), and since the available K currents in this dendritic location have small conductances relative to the NMDA conductances, we have added a leak conductance in the same order of magnitude as the NMDA current to be able to obtain phase curves in the bistable regime (Lazarewicz et al., 2006; Shoemaker, 2011; Sanders et al., 2013). As described in the Results section, the y-axes of the phase plot show $C_{m}\overset{•}{V}$, i.e., they show the current:


$$\begin{array}{l} I=-\sum I_{\text{ion}}-I_{\text{NMDA}}-I_{\text{leak}}. \end{array}$$

### 2.6. Procedure for fitting the gating function parameters from experimental data

We follow the procedure presented in Jahr and Stevens (1990b) to fit the parameters α and η we list in Table 2. We first digitize the data using WebPlotDigitizer (Rohatgi, 2022) for current-voltage relationships for the available Mg^2+^ concentration from the figures in the articles, from which we then derive the conductance-voltage relationships. When converting the current amplitudes to conductance, the different voltage reversal potential across different experimental conditions was taken into account. Specifically, following Ohm's Law, $g_{\text{NMDA}}=\frac{I_{\text{NMDA},\text{ peak}}}{V_{\text{holding}}-V_{\text{rev}}}$. Conductance-voltage relationships were normalized with the maximal conductance of each curve, which is the point obtained for the highest applied voltage (usually +50 mV or above) when all of the NMDARs are free from Mg^2+^. Finally, the normalized conductance-voltage relationships are fitted to Equation (4) using the Python function scipy.optimize.curve_fit.

## 3. Results

### 3.1. Thresholded glutamate spillover causes robust all-or-none behavior of plateau potentials

**Figure 2:**
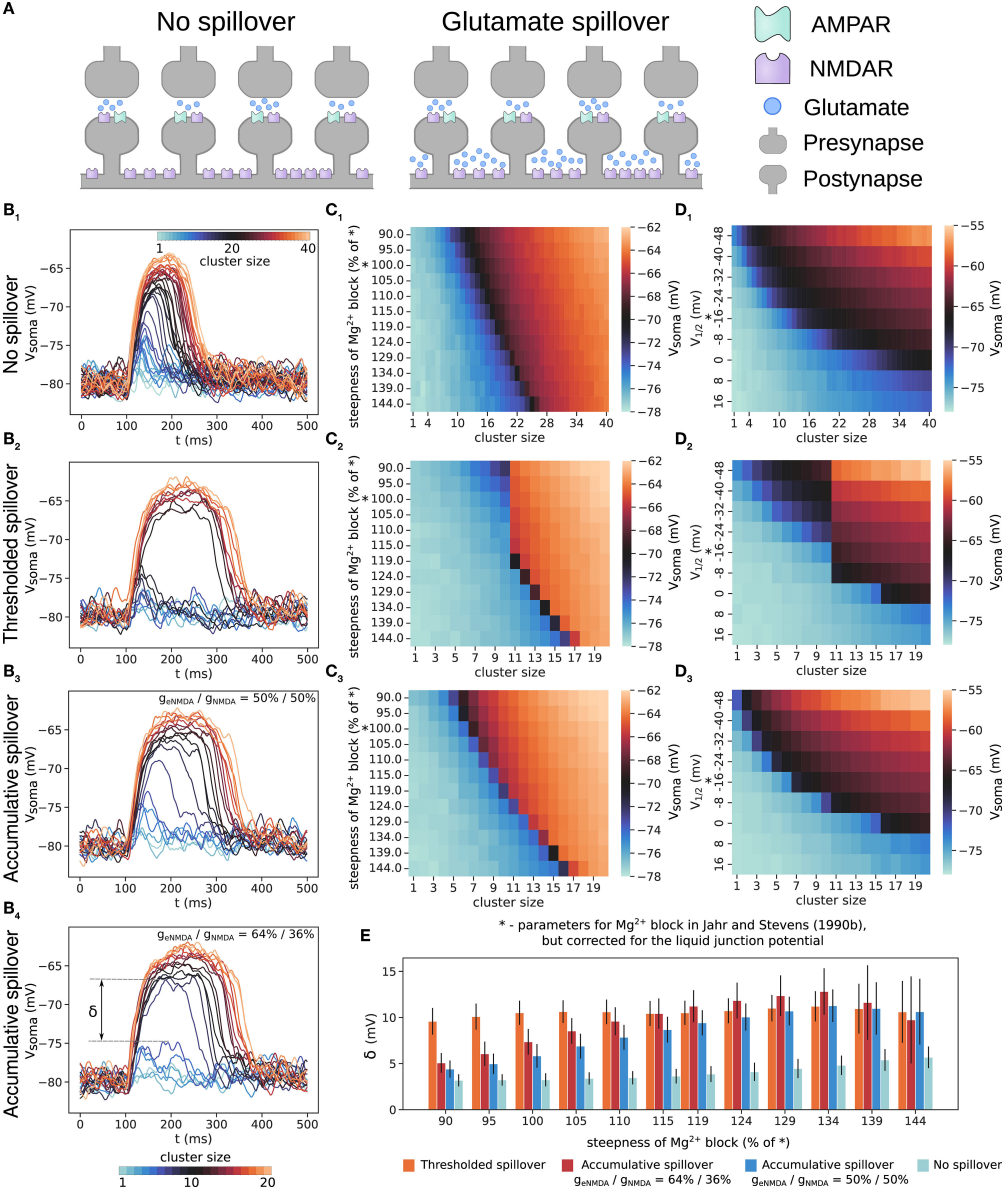
Plateau potentials evoked with and without glutamate spillover. **(A)** Illustration of glutamate spillover vs. no spillover. **(B)** Somatic voltage traces of plateau potentials evoked by a cluster of increasing size from 1 to 40 synapses for the model with no spillover **(B**_1_**)** and from 1 to 20 synapses for the models with: thresholded spillover **(B**_2_**)**, accumulative spillover with equal proportions of NMDAR and eNMDAR conductance **(B**_3_**)**, and accumulative spillover with a larger proportion of eNMDAR conductance (64% of total NMDAR conductance); **(B**_4_**)**. **(C)** Amplitude of somatic depolarization as a function of cluster size and steepness of the gating function for the models with: no spillover **(C**_1_**)**, thresholded spillover **(C**_2_**)**, accumulative spillover with equal proportions of NMDAR and eNMDAR conductance **(C**_3_**)**. **(D)** Amplitude of somatic depolarization as a function of cluster size and position of the gating function along the x-axis (determined by varying the parameter η according to η = *e*^*x*^, for *x*∈{−3, −2.5, …, 2}). **(D**_1_**–D**_3_**)** show results for the same models as in **(C**_1_**–C**_3_**)**. Values in the heatmaps are averages over 50 trials of clustered synaptic inputs elicited in the same dendrite as for the panels in **(B)**, except for **(C**_1_**, D**_1_**)**, which are averages over 30 trials. The “*” on the y-axes in **(C, D)** indicates the gating function parameters as in Jahr and Stevens (1990b), but corrected for the liquid junction potential. **(E)** The maximal difference between two consecutive voltage traces, δ, also called the size of the voltage jump, for the four models in **(B)**. Large jumps indicate all-or-none plateau potentials, and small jumps are consistent with graded NMDA spikes/plateau potentials. δ for no spillover was calculated taking every second voltage trace (steps of 7 nS in NMDA conductance), so that it is comparable to the δ calculated for **(B**_3_**, B**_4_**)**. Results are averages over 50 trials and 11 different dendrites, with plateaus elicited at approximately the same distance from the soma, except for the model with no spillover, where they are averages over 30 trials in 11 different dendrites. Error bars represent standard deviation.

In Figure 2, we compare the plateau potentials generated by a cluster of increasing size with the two spillover models and without glutamate spillover and with the originally reported parameters for the Mg^2+^ block but corrected for the value of the junction potential (i.e., the parameters found in Jahr and Stevens, 1990b but corrected as in Ecker et al., 2020). The somatic voltage traces in Figures 2B_1_, B_2_ show that plateau potentials generated without glutamate spillover exhibit a graded increase in amplitude, whereas those generated with thresholded glutamate spillover exhibit an all-or-none response. As described in the Methods, in order to compare the same total NMDAR conductance between the two scenarios, the model with no spillover has a twice larger cluster size than the models with glutamate spillover.

We also varied the steepness of the gating function by varying the parameter α in Equation (4), and the amplitude of the plateau potentials thus generated is shown as heatmaps in Figures 2C_1_, C_2_. The all-or-none behavior can be seen as a sharp jump in the colors of the heatmaps for a small increase in cluster size (i.e., stimulus strength for evoking a plateau). Without glutamate spillover the all-or-none behavior is present only for steep sigmoidal curves, whereas with thresholded glutamate spillover, it is always present. (Steeper gating functions require more excitation to overcome the Mg^2+^ block in both cases). As described above, we also estimated the gating function parameters for available experimental data (Table 2, sigmoid curves plotted in Supplementary Figure 2). Most of these gating functions are more shallow than the originally reported one by Jahr and Stevens (1990b), and result in graded NMDA potentials in the model without spillover, but still provide all-or-none plateau potentials in the model with glutamate spillover. When varying the parameter η, which shifts the gating function along the x-axis, the same results regarding the all-or-none behavior are observed for the models with thresholded spillover and without glutamate spillover as when varying the parameter α (Figures 2D_1_, D_2_). Shifting the gating functions to very depolarized values disables the generation of plateau potentials, as seen from the heatmaps in Figures 2D_1_, D_2_. In Figure 2E, we compare the maximal difference between the somatic voltage amplitudes of two consecutive voltage traces, termed the “size of the jump,” δ (indicated in Figure 2B_4_). In our view, the size of the jump, δ, is essentially proportional to the system's nonlinearity. For example, a large sudden jump (≈10 mV) indicates all-or-none behavior, whereas small jumps (of a few mV) would indicate graded potentials. In our model, thresholded glutamate spillover produced voltage jumps of 8–12 mV, while no spillover produced jumps of ≈3 mV, typically (Figure 2B_1_).

In addition, the somatic amplitude of plateau potentials generated with thresholded spillover and without glutamate spillover decays with increasing the cluster distance from the soma as shown in Supplementary Figure 3 (Major et al., 2008).

### 3.2. Importance of extrasynaptic NMDAR activation for all-or-none plateau potentials: accumulative vs thresholded spillover

Physiologically relevant parallel fiber stimulation protocols cause gradual accumulation of glutamate with each stimulation pulse in cerebellar extrasynaptic space (Okubo et al., 2010). To explore the effects of such more gradual spillover, we performed simulations where each activation of spine NMDARs also activates the corresponding eNMDARs in the dendritic shaft under the spine after a delay, without the need to first reach a glutamate threshold. (the accumulative spillover described in Section 2).

The results of these simulations show that accumulative glutamate spillover exhibits plateau potential behavior which is between the behavior without spillover and with thresholded glutamate spillover (Figures 2B_3_, C_3_, D_3_). Shallow gating functions provide graded plateau potentials, but all-or-none behavior arises much sooner for increasing steepness than the in case without spillover. With thresholded spillover, clusters of up to 10 synapses only access the spine NMDAR conductance, and clusters with more than 10 synapses activate both NMDAR and eNMDAR conductances, thus producing the large voltage jump before and after spillover (Figure 2B_2_). With accumulative spillover, eNMDARs are activated with a short delay after each NMDAR activation, giving rise to the more graded increase in plateau potential amplitude, as well as duration (Figure 2B_3_). Moreover, for a larger proportion of eNMDAR conductance in the total conductance, the behavior is more all-or-none (Figures 2B_4_, E). This suggests that there may be particular conditions under which all-or-none plateau potentials appear and that plateau potential behavior might vary among brain regions.

### 3.3. Spillover induces all-or-none plateau potentials by facilitating the switch to bistable or self-triggering regime

**Figure 3:**
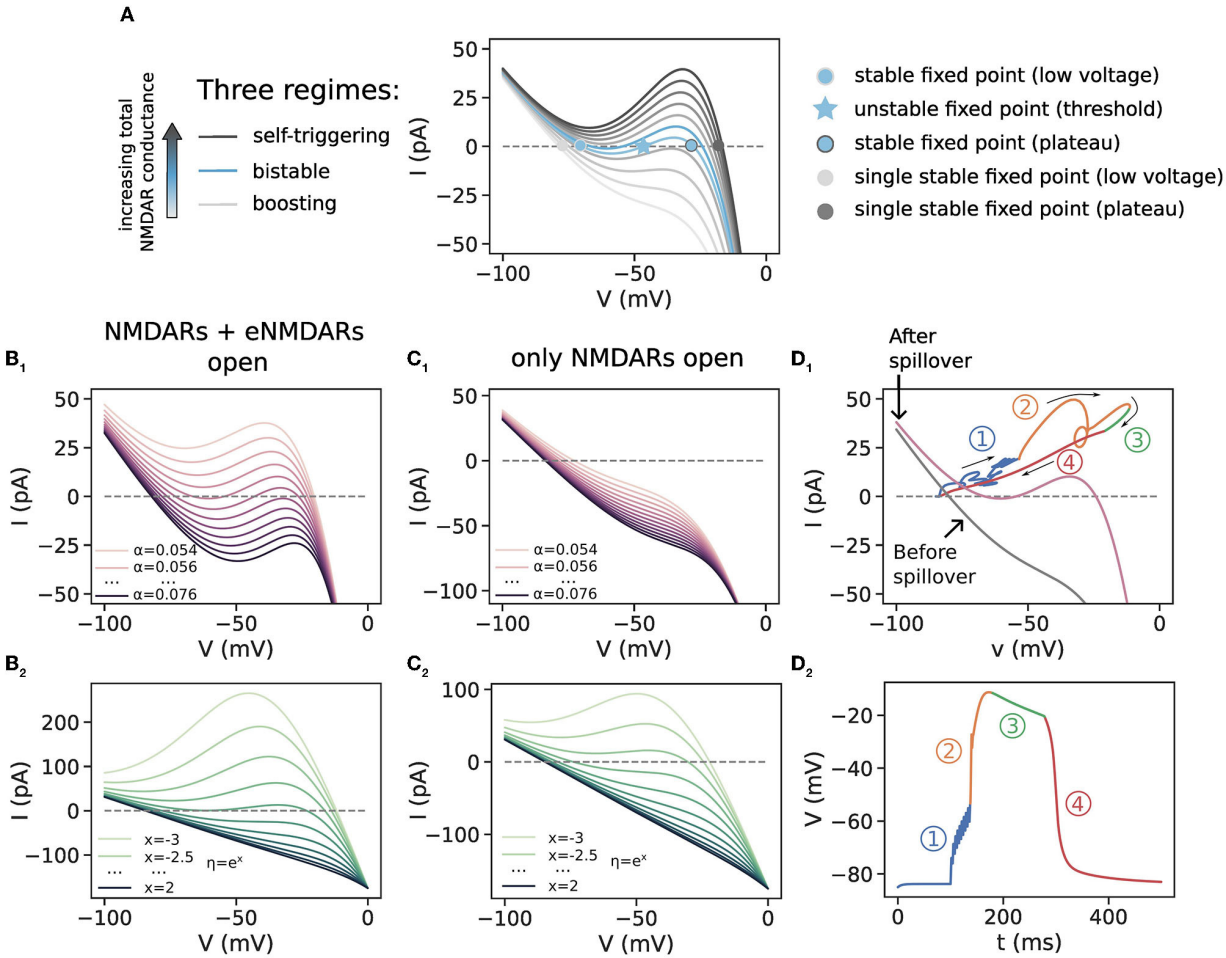
Phase plot analysis for a single membrane compartment. **(A)** Illustration of the three regimes in the phase plot which are obtained for varying levels of NMDAR conductance. This panel is a reproduction of the plot in Supplementary Figure 4 in Major et al. (2008) (note the inverted direction of the y-axis in Major et al., 2008). As the total NMDAR conductance increases, the phase curve moves through the boosting, bistable and self-triggering regions, and as the NMDARs close, the phase curve moves back in the other direction. The boosting and self-triggering regime each have a single stable fixed point, corresponding to resting and plateau voltage, respectively. The bistable regime has three fixed points, two of which are the stable resting and plateau voltage levels. The middle, unstable fixed point is the voltage threshold that needs to be crossed for a plateau potential to occur. **(B**_1_**, B**_2_**)** Phase plots for varying levels of α and η, respectively. The total NMDAR conductance here models both NMDARs and eNMDARs activated. **(C**_1_**, C**_2_**)** Phase plots for the same ranges in α and η but for half of the total NMDAR conductance in **(B**_1_**, B**_2_**)**, mimicking the situation before thresholded glutamate spillover when only synaptic NMDARs are activated. α ∈{0.054, 0.058, …, 0.094} and η = 0.38 in **(B**_1_**, C**_1_**)** and α= 0.062 and η given by η = *e*^*x*^, for *x*∈{−3, −2.5, …, 2} in **(B**_2_**, C**_2_**)**. Lighter colors correspond to lower values. **(D**_1_**)** A trajectory of a plateau potential plotted on the phase plot. Two-phase curves, for the situations before and after spillover, are plotted in gray and purple, respectively. The four regions depict the four phases of the plateau potential in **(D**_2_**)**. Thin arrows indicate the direction of the trajectory through the phase plot. **(D**_2_**)** The dendritic voltage of the plateau potential whose trajectory is plotted in **(D**_1_**)**. This plateau potential is generated with a cluster of 10 synapses with weight *w* = 0.45 in order to reach the glutamate threshold with 10 synapses and cause glutamate spillover. 1, subthreshold excitation; 2, suprathreshold excitation; 3, plateau phase; 4, downward, hyper-polarizing phase. α = 0.062, η = 0.38.

To explain the results in the previous section, we use phase plot analysis of the membrane voltage calculated for a single membrane compartment (Figure 3). Phase plot analysis reveals the role of different membrane conductances in generating nonlinear behavior such as plateau potentials. In particular, it has been found that apart from the nonlinear, voltage-dependent excitatory NMDA current, an opposing, shunting current is also required acting at least on the longer timescale of NMDA currents. This role can be played by K_ir_ channels, GABA_B_ currents, a passive leak current, and/or an axial current diverting charge to the rest of the neuron (Lazarewicz et al., 2006; Shoemaker, 2011; Sanders et al., 2013).

We begin with a short description of how to read the phase plots (Figure 3A). A voltage phase plot normally contains voltage on the x-axis and the rate of voltage change (the time derivative of the voltage, $\overset{•}{V}$) on the y-axis. We have instead plotted the membrane current on the y-axis, which is simply a linear rescaling of $\overset{•}{V}$ with the membrane capacitance, $I=C_{m}\overset{•}{V}$. The intersections of the phase curve with the x-axis correspond to fixed points in the voltage dynamics ($\overset{•}{V}=0$). Since our single compartment is a one-dimensional system (voltage is the only independent state variable), a point in the phase plot can move only along the phase curve. Initial voltage points that correspond to positive values of the phase curve ($\overset{•}{V}>0$) will get increased in time, whereas those corresponding to negative values get decreased. Voltage change stops when a point on the phase plot reaches a stable fixed point (where $\overset{•}{V}=0$). Points near unstable fixed points are repelled away from them. The trajectory from the multicompartment SPN model shown in Figure 3D_1_, on the other hand, can take on any values in the phase plot. This is because in the recorded dendritic compartment, the gating variables of the ion channels are also time-varying, i.e., they are additional state variables in the model. We note that the phase plot is different from the commonly used and similarly named I–V plot, which can be found, for example, in the aforementioned articles measuring macroscopic NMDA currents.

Depending on the total NMDAR conductance in a dendritic region, NMDARs can exhibit three types of nonlinear dynamics, as shown in Figure 3A: boosting, bistable, and self-triggering, of which the latter two correspond to plateau potentials (Schiller and Schiller, 2001). Boosting behavior occurs for small or moderate amounts of total NMDAR conductance which is not enough to trigger plateau potentials. In this case other excitatory inputs are boosted in amplitude by activating the longer-lasting NMDA potentials. The phase curve has one stable fixed point to the left of the diagram at a low voltage. Bistable behavior is exhibited when the phase curve has an inverted-N shape with three interceptions with the x-axis (fixed points), which happens for larger amounts of total NMDAR conductance. In this case a plateau potential is generated for an excitation that exceeds the voltage threshold determined by the middle (unstable) fixed point. Such an excitation causes a fast excursion in the phase plot to the right (higher) stable fixed point, which causes the all-or-none behavior (region 2 in the phase plot in Figure 3D_1_, which corresponds to the upward swing in the corresponding dendritic voltage in Figure 3D_2_, and animation in Supplementary data). Even larger total NMDAR conductance gives rise to the so-called self-triggering behavior. In this case the phase curve, now situated above the bistable region, is inverted-N-shaped and has one stable fixed point to the right of the diagram which corresponds to high voltage. In this case, any excitation produces a plateau potential by carrying the voltage to the single fixed point. Thus, as the total NMDAR conductance increases with successive activations of synapses in a cluster, the phase curve sweeps potentially across all three regions in the phase plot. On the other hand, closing of the NMDARs moves the phase curve back in the other direction, collapsing the inverted-N shape back toward a straight line and terminating the plateau potential with the voltage dropping to the low-voltage, single stable fixed point, which corresponds to the resting voltage.

To elicit plateau potentials, the total NMDAR conductance must be enough to situate the phase curve in the bistable or self-triggering region (the threshold level of NMDAR conductance being at the border between the boosting and the bistable region). An example of a plateau potential generated with thresholded glutamate spillover, with the corresponding phase plot trajectory, is given in Figures 3D_1_, D_2_. The gray phase curve in Figure 3D_1_ is the one obtained when only synaptic NMDAR conductance is activated, whereas the purple curve represents the total NMDAR conductance with spillover, when eNMDARs are activated. After the subthreshold excitation by nine synaptic inputs (region 1), the 10th input causes spillover and the dendritic voltage crosses the threshold determined by the unstable fixed point. This initiates the self-sustaining suprathreshold excitation (region 2), which is followed by the plateau region (region 3). The end of the extrasynaptic transmitter pulse starts the downward, hyper-polarizing phase (region 4).

To examine the role of the gating function parameters α and η, we constructed phase plots with varying α and η. Figures 3B_1_, B_2_ are a model of the total NMDAR conductance when both NMDARs an eNMDARs are activated during spillover, as well as the case when all of this total conductance is situated in the spines of the equivalent model without spillover (Figure 2B). Varying the steepness of the Mg^2+^ block shifts the phase curve vertically, and all three phase plot regimes may be obtained (Figure 3B_1_). Shallow sigmoidal curves have only one fixed point and are in the self-triggering region, whereas steep sigmoidal curves have three fixed points and are in the bistable region. Very steep sigmoidal curves are in the boosting region. In Figures 3C_1_, C_2_, on the other hand, the total NMDAR conductance is halved, to model only the synaptic NMDAR conductance accessible before thresholded spillover. In this case, all phase curves for the single membrane compartment are in the boosting region, regardless of the gating function steepness.

These results from the phase plots can explain the plateau potential behavior with and without spillover. Total NMDAR conductance increases with increasing cluster size—larger clusters have greater total NMDA conductance (Figure 2B, cluster size). When there is no glutamate spillover, all-or-none behavior is observed only for steep sigmoidal curves, since the corresponding phase curves are in the bistable regime, while for the shallow sigmoidal curves, all synaptic clusters of increasing size produce graded excitations as in Figure 2B_1_, because the phase curve is in the self-triggering region with a single, high-voltage fixed point (Figure 3B_1_). Graded excitations trigger NMDA spikes first and plateau potentials later, as the total NMDA conductance increases. On the other hand, in the model with glutamate spillover, activating only the synaptic NMDA conductance (extrasynaptic NMDARs are inactive) is insufficient to elicit plateau potentials (the phase curve is most likely in the boosting region as in Figure 3C_1_). When spillover occurs, the activation of the eNMDARs provides enough NMDA conductance to elicit a plateau potential. This shifts the phase curve from the boosting to the self-triggering region for shallow gating functions and to the bistable region for steep gating functions. The duration of the phase curve in both of these regions is significantly prolonged due to glutamate spillover. This shift (and inversion) of the phase curve occurs suddenly because, as described in the Methods section, in thresholded glutamate spillover all the eNMDAR conductance is simultaneously activated. For shallow sigmoidal curves, it is this sudden inversion that causes the all-or-none plateau potential behavior. For steep sigmoidal curves, the voltage excitation also needs to be higher than the voltage threshold for a plateau to occur. In this way, glutamate spillover causes all-or-none plateau potentials for a wide range of gating function steepness. Accumulative spillover, on the other hand, is very similar to no spillover. Each successive synaptic activation gradually increases the total NMDAR conductance, slowly moving the phase curve across the phase plot.

Varying the parameter η shifts the gating function along the x-axis. Shifting the function to higher potentials flattens (linearizes) the phase curve. While all three regimes can be obtained with various values of η, shifting the gating function to higher potentials results in only boosting behavior and disappearance of plateau potentials both with and without glutamate spillover (Figures 2D_1_–D_3_, 3C_1_, C_2_).

### 3.4. Long glutamate duration drives all-or-none plateau potentials

**Figure 4:**
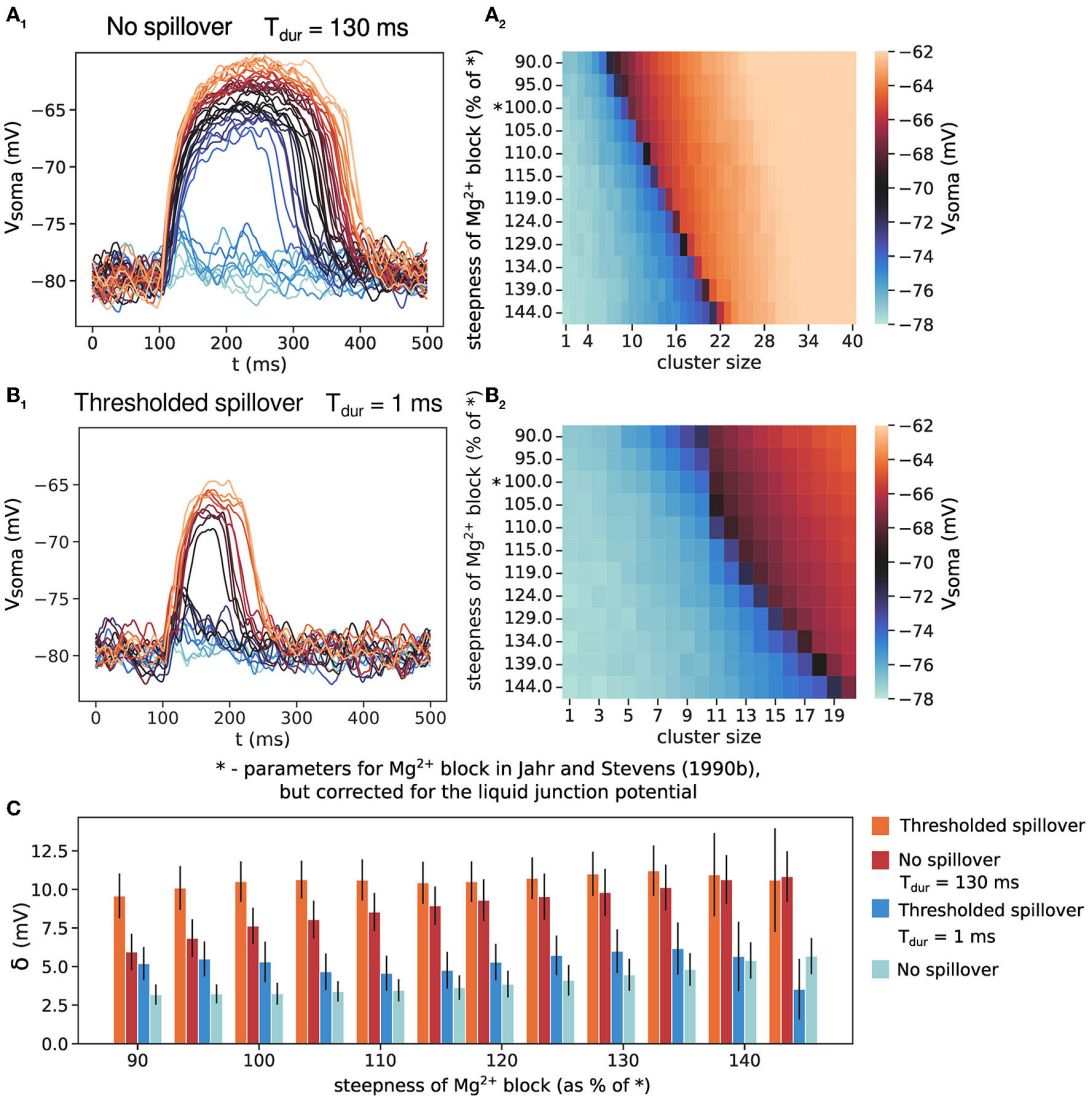
The effect of transmitter pulse duration, T_dur_, on the all-or-none behavior of plateau potentials. **(A**_1_**)** No glutamate spillover but long transmitter pulse duration produces all-or-none plateau potentials. Cluster size up to 40 synapses. **(A**_2_**)** Somatic amplitude of plateau potential when varying the steepness for the model in **(A**_1_**)**. **(B**_1_**)** Thresholded glutamate spillover and short transmitter pulse in the extrasynaptic space produces NMDA potentials with a small all-or-none jump. Cluster size up to 20 synapses. **(B**_2_**)** Somatic amplitude of plateau potential when varying the steepness for the model in **(B**_1_**)**. The “*” on the y-axes in **(A**_2_**, B**_2_**)** indicates the gating function parameters as in Jahr and Stevens (1990b), but corrected for the liquid junction potential. **(C)** The size of the voltage jump, δ, for the scenarios in **(A, B)**. Results for thresholded spillover and no spillover from Figure 2E are added for ease of comparison. Results are averages of 30 trials and 11 different dendrites for **(A**_1_**)** and 50 trials and 11 different dendrites for **(B**_1_**)**, with clusters positioned at approximately the same distance from the soma. Error bars represent standard deviation.

We verify that it is indeed the length of glutamate spillover that causes all-or-none plateau potential behavior by manipulating the duration of the transmitter pulse (the parameter T_dur_, Figure 4). As explained in the Methods section, T_dur_ for the synapses onto spines is much shorter than T_dur_ for eNMDARs. Increasing T_dur_ in the case without spillover results in all-or-none plateau potential behavior. Again, increasing NMDAR conductance by increasing cluster size moves the stable fixed point further to the right in the phase plot in Figure 3A. When entering the bistable regime, the all-or-none jump appears in Figure 4A_1_, and lasts because the increased T_dur_ keeps the phase curve in the bistable regime for a longer time. On the other hand, decreasing the eNMDAR T_dur_ in the case with spillover produces rather graded plateau potentials with much smaller voltage jumps (Figure 4B_1_). The all-or-none behavior in Figure 4A_1_ and graded behavior in Figure 4B_1_ are also evident in the sharp and gradual color transitions in the respective heatmaps in Figures 4A_2_, B_2_, as well as in the comparison of the voltage jump, δ in Figure 4C.

### 3.5. Low extrasynaptic glutamate concentration can produce all-or-none plateau potentials

**Figure 5:**
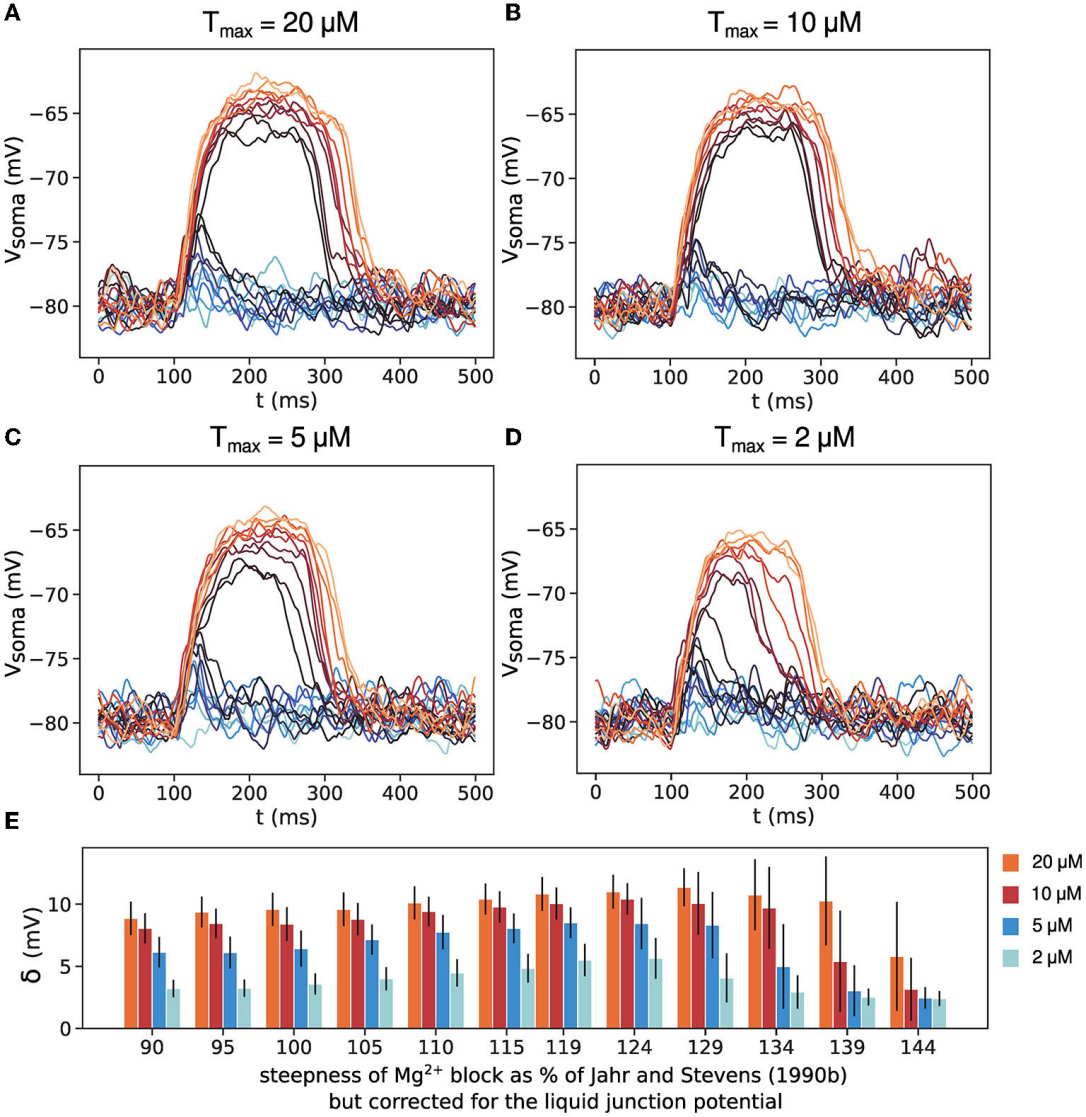
The effect of low extrasynaptic glutamate concentration. **(A–D)** Somatic voltage traces for thresholded spillover and 20, 10, 5, and 2 μM extracellular glutamate concentration, T_max_. **(E)** Size of voltage jump δ for the four values of T_max_. Results are averages of 50 trials in 11 different dendrites with clusters positioned at approximately the same distance from the soma. Error bars represent standard deviation.

The extrasynaptic glutamate concentration (the parameter T_max_ for eNMDARs) used in the simulations above is 200 μM, an order of magnitude higher than the measurements reported in Okubo et al. (2010). NMDARs have high-affinity for glutamate, in the low-micromolar range, so they should be able to respond to low glutamate concentrations. We have tested the thresholded spillover model with lower values for extracellular glutamate concentration (the parameter T_max_) and the results are shown in Figure 5. Concentrations of 10 and 20 μM, significantly higher than the NMDAR dissociation constant, *K*_*d*_ = 2.5 μM, produce all-or-none plateau potentials (Figures 5A, B). For concentrations of 5 μM, the size of the voltage jump is reduced, although the plateau potentials retain their all-or-none quality. Concentrations comparable to the NMDAR *K*_*d*_ do not activate the eNMDARs fully and produce graded plateau potential amplitude (Figures 5D, E).

### 3.6. All-or-none behavior of plateau potentials enables nonlinear computation, such as solving the nonlinear feature binding problem

Linearly non-separable tasks, an example of which is the nonlinear feature binding problem (NFBP) cannot be solved by the perceptron, a computational unit with a single nonlinearity, and require a network of such artificial neurons. As such, these tasks are traditionally used as a benchmark for the computational capabilities of a computational unit. Possessing dendritic nonlinearities in addition to the somatic nonlinearity, single biological neurons have been shown to be equivalent to at least a two-layer artificial neural network (Poirazi et al., 2003) and should, in principle, be able to solve such tasks (Tran-Van-Minh et al., 2015). Although it is not known whether the NFBP is solved by single neurons *in vivo*, and whether it is a relevant task for any brain region, human cortical layer 2/3 pyramidal neurons have been found to possess calcium-mediated dendritic spikes whose voltage dependency enables the dendrite exhibiting them to solve the exclusive OR (XOR) problem in simulations (Gidon et al., 2020), and the XOR problem is related to the NFBP (Cazé et al., 2013). These dendritic nonlinearities occur in the apical dendrites, while the SPN dendrites correspond to basal dendrites in pyramidal neurons, where NMDA nonlinearities occur. We, therefore, use the NFBP as a benchmark to demonstrate how all-or-none plateau potentials enable a single neuron to solve this task.

**Figure 6:**
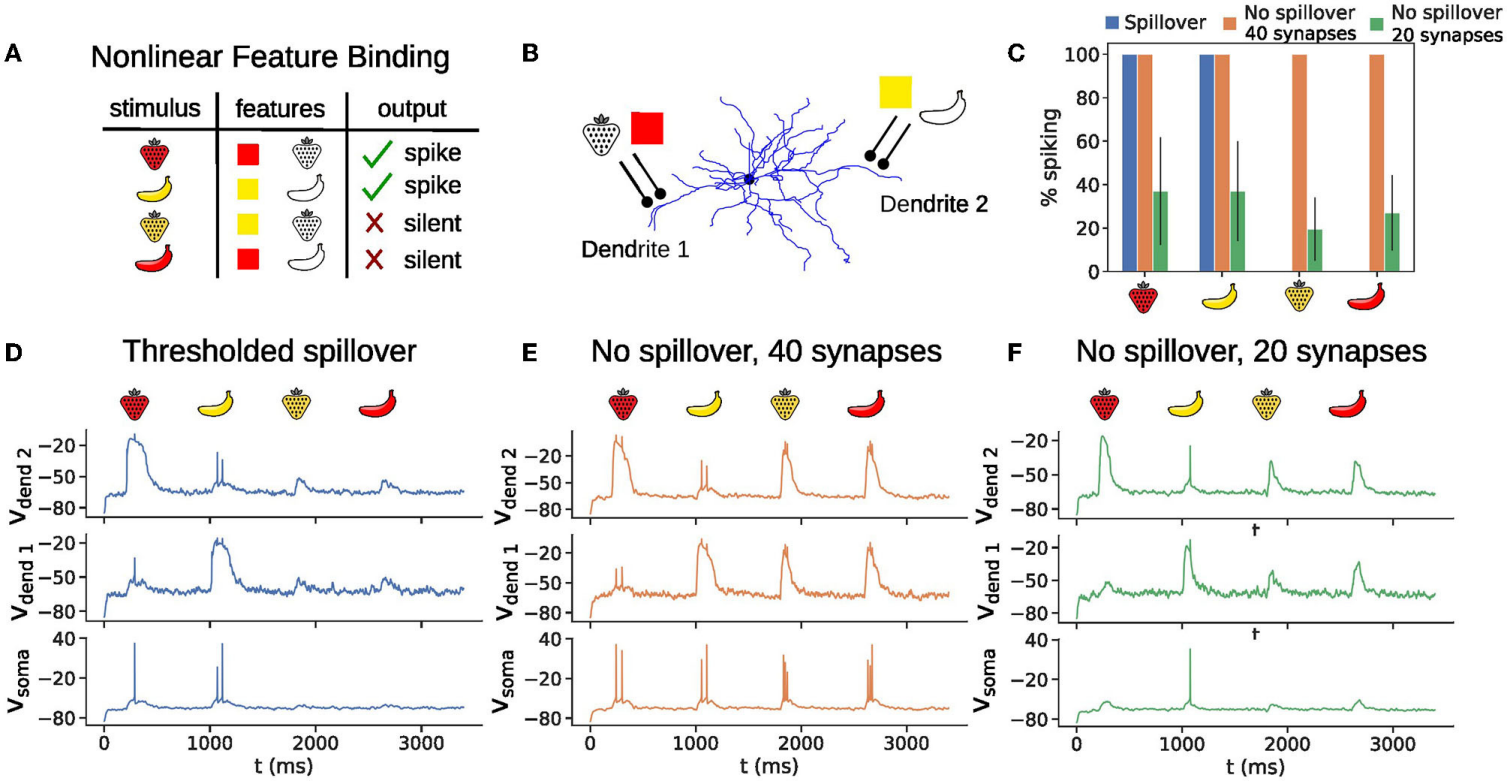
All-or-none plateau potential behavior is useful for solving the nonlinear feature binding problem (NFBP). **(A)** The NFBP illustrated with an example from visual feature binding. **(B)** An arrangement of synapses in clusters that could solve the NFBP. A black circle on the figure represents 10 synapses in the thresholded spillover model and no spillover model containing clusters of 20 synapses **(D, F)**, and 20 synapses in the no spillover model containing clusters of 40 synapses **(E)**. **(C)** Somatic spiking as a percentage for 40 presentations of each stimulus for the thresholded spillover model and the no spillover model with clusters of size 20 and 40 synapses on the NFBP. Error bars represent standard deviation. Note that with thresholded spillover the task performance is 100%, and that no spillover with 40 synapses always elicited somatic firing, resulting in zero-sized error bars in these cases. **(D–F)** Example voltage traces for each stimulus presentation in the scenarios with thresholded spillover **(D)**, no spillover with a cluster of 40 synapses **(E)**, and no spillover with clusters of 20 synapses **(F)**. The background noise in these examples is increased (compared to the results in Figures 2, 4, 5), so that the neuron spikes with additional clustered inputs representing shape and color. Simulations were repeated for five pairs of dendrites.

We illustrate the NFBP with an example from visual feature binding (Figure 6A). A stimulus has two features (shape and color), each of which can have two values (strawberry or banana for the shape, and red or yellow for the color), for a total of four possible combinations. Each stimulus excites the neuron with the same amount of excitation, on average. The task consists of responding to two of the feature combinations (the relevant stimuli) with a somatic spike and remaining silent for the other two (irrelevant stimuli). This means that the same amount of total excitation should be processed differently, and the plateau potentials offer a natural solution to the task: the synapses for the relevant stimuli should be clustered in separate dendrites (Figure 6B), and should elicit a plateau potential upon arrival of a relevant stimulus which will drive somatic spiking. Irrelevant stimuli would activate only half of the cluster and should not elicit a plateau potential (Tran-Van-Minh et al., 2015). This setup with thresholded glutamate spillover provides perfect performance on the NFBP (Figures 6C, D). Without spillover, the graded amplitudes of the plateaus cause somatic spiking for the irrelevant stimuli as well, and the neuron cannot perform this task (Figures 6C, E, F).

## 4. Discussion

In this computational study we investigated how to generate robust all-or-none dendritic plateau potentials using striatal projection neuron models as a test case. We also illustrated how such plateau potentials can enhance computation. Dendritic nonlinearities in the form of NMDA spikes and plateau potentials offer an enhanced ability for dendritic computation compared to the linear summation of excitatory inputs (Poirazi et al., 2003; Lavzin et al., 2012; Xu et al., 2012; Tran-Van-Minh et al., 2015; Kumar et al., 2018). By summating co-activated clustered inputs supralinearly, they can allow for the preferential detection of certain input patterns or combinations of inputs. For example, in the mouse barrel cortex, layer 4 spiny stellate neurons generate local and global multi-branch NMDA spikes that contribute substantially to the angular tuning of these neurons (Lavzin et al., 2012). In addition, distal dendrites of layer 5 pyramidal neurons in the barrel cortex might integrate correlated sensory and motor information via dendritic nonlinearities in order to produce a signal related to object localization during active sensing tasks (Xu et al., 2012). Dendritic plateau potentials are also essential processes for triggering synaptic plasticity both *in vitro* and *in vivo* (Gambino et al., 2014; Cichon and Gan, 2015; Brandalise et al., 2016). For example, in the somatosensory cortex, rhythmic sensory whisker stimulation can induce synaptic long-term potentiation (LTP) in layer 2/3 pyramidal cells, which is triggered by plateau potentials generated through the cooperative activity of the intracolumnar lemniscal and thalamocortical paralemniscal synaptic circuitry in the absence of somatic spiking (Gambino et al., 2014). Additionally, different motor learning tasks induce dendritic calcium spikes on different apical tuft branches of layer 5 pyramidal neurons in the mouse motor cortex (Cichon and Gan, 2015). These task-related, branch-specific calcium spikes cause long-lasting potentiation of postsynaptic dendritic spines active at the time of spike generation, suggesting a role for dendritic nonlinearities in storing new information without disrupting previously acquired memories (Cichon and Gan, 2015). Lastly, dendritic nonlinearities have been suggested to enable a neuron to solve linearly non-separable tasks such as the NFBP (Tran-Van-Minh et al., 2015).

Experimentally evoked plateau potentials *in vitro* display all-or-none behavior, and this feature is not easily nor robustly captured when modeling. A critical ingredient for the all-or-none property is the sigmoidal shape of the Mg^2+^ block voltage dependence, described by the gating function in Equation (4). The many NMDAR isoforms show different sensitivity to Mg^2+^, which results in a range of possible shapes of the sigmoid gating function in terms of its steepness and the voltage required to overcome the Mg^2+^ block. We have studied how the variability of these gating function properties affects the all-or-none plateau potential behavior. We found that the all-or-none property is very sensitive to the steepness of the gating function. Shallow gating functions are in the self-triggering region of the phase plot and result in graded plateau potential amplitudes for increasing cluster size, whereas steep gating functions are in the bistable region and result in all-or-none plateau potentials (Figures 2B_1_, B_2_, 3B_1_). Importantly, including glutamate spillover in the models provides a robust all-or-none quality of the plateau potentials for the whole tested range of Mg^2+^ block steepness.

Almost all the gating functions we fitted to available experimental data are more shallow than the originally reported sigmoidal curve by Jahr and Stevens (1990b), which already results in graded plateau potential amplitude in the SPN model (Figure 2B; Table 2). Conversely, all of the studies that model plateau potentials, except for Ecker et al. (2020), that correct for the omitted junction potential in Jahr and Stevens (1990b), have increased the steepness of the originally reported gating function without explicitly stating a reason for it. As shown in Figure 3B_1_, increasing the steepness of the gating function can move the phase curve in the bistable region, allowing to robustly generate all-or-none plateau potentials, thus offering an explanation for the steeper gating functions in modeling studies. Moreover, in light of the natural variability in the Mg^2+^ block gating function, the question remains as to how SPNs generate the all-or-none plateau behavior robustly in experiments with increasing stimulation. According to the results above, glutamate spillover is predicted to be a part of the plateau potential generation mechanism, providing robust all-or-none behavior across a wide range of slopes of the Mg^2+^ block gating function.

Whether, *in vivo* plateau potentials are graded or all-or-none is not known. However, in Gambino et al. (2014) for example, robust, seemingly all-or-none plateau potentials are evoked with co-activation of the intracolumnar lemniscal and thalamocortical paralemniscal pathways, while lemniscal pathway activation alone significantly reduces the probability of evoking plateau potentials. In this study we have illustrated the importance of such all-or-none plateau potentials in a commonly used benchmark task, the NFBP, showing that graded plateau potentials cannot perform this task compared to the perfect performance with all-or-none plateau potentials. It is not known whether the NFBP is solved by single neurons in any brain region on a regular basis, but plateau potentials, whether used for the NFBP or in the detection/representation of single features, have been suggested to prepare the neuron to spike in a robust fashion as a response to the particular plateau-evoking input (Antic et al., 2018). *In vitro*, distally evoked plateau potentials in the basal dendrites of pyramidal neurons typically do not evoke somatic spiking by themselves (Milojkovic et al., 2004; Major et al., 2008; Gao et al., 2021), and neither do plateau potentials in SPNs (Plotkin et al., 2011; Du et al., 2017). This corresponds to the situation we modeled in Figures 2, 4, 5. However, in the presence of additional inputs or background noise, plateau potentials drive a robust somatic response to the particular input pattern that evoked them, as we exemplify in Figure 6. According to the predictions from this study, the prolonged activation of extrasynaptic NMDARs is critical for robust plateau potential generation.

Despite important advances such as the confirmation of glutamate spillover *in vivo*, the confirmation of its role in neuronal signaling in the cerebellum, and reports of extracellular glutamate concentrations in experimentally evoked spillover in brain slices in the low micromolar ranges, many important details await further studies (Szapiro and Barbour, 2007; Okubo et al., 2010; Papouin and Oliet, 2014; Rusakov and Stewart, 2021). Some prominent missing details are: (i) the lifetime of glutamate in the extrasynaptic and perisynaptic spaces; (ii) the conditions under which possible astrocytic glutamate release and/or reversal of glutamate uptake occurs; as well as (iii) control of eNMDAR activation by the co-agonist glycine or d-serine (Malarkey and Parpura, 2008; Papouin and Oliet, 2014). Assuming high NMDAR affinity for glutamate, we have shown that 10–20 μM of extracellular glutamate can produce all-or-none plateau potentials if glutamate persists in the extracellular space for long durations, indicating that physiological concentrations may play a large role in signal integration. Recent findings that LTP boosts glutamate spillover by initiating withdrawal of perisynaptic astroglial processes suggest dynamic regulation of the synaptic and perisynaptic environment, including eNMDAR activation, for the purposes of signal processing and learning (Henneberger et al., 2020; Rusakov and Stewart, 2021). Facilitation of the prolonged eNMDAR activation would, in turn, increase the probability that the already strengthened synapses can evoke a robust dendritic plateau potential.

One direction for future work would be to investigate how the distinct gating functions of various NMDAR isoforms affect dendritic computations in different neuron types in the brain, as well as to further quantify the contribution of extrasynaptic NMDA receptors for these computations (Zhou et al., 2015). Another direction for future work would be to develop more detailed diffusion models of glutamate concentration activating extrasynaptic NMDARs for a quantitatively more precise understanding of the phenomenon in particular synapse types. However, since NMDARs have high affinity for glutamate, even low extrasynaptic glutamate concentrations also generate robust plateau potentials for a prolonged extrasynaptic glutamate signal (as shown in Figure 5). The synaptic model that we used assumes fixed transmitter concentration for the duration of the transmitter pulse both in the synaptic cleft and in the extrasynaptic space. Glutamate in the synaptic cleft rapidly reaches its peak concentration and is also quickly cleared due to diffusion and reuptake (Clements et al., 1992). In contrast to that, extrasynaptic glutamate dynamics is likely more variable. Under conditions of temporary saturation of astrocytic glutamate uptake and possibly Ca^2+^-dependent astrocytic glutamate exocytosis and/or reverse operation of astrocytic glutamate transporters (Malarkey and Parpura, 2008), extrasynaptic glutamate concentration could remain elevated long enough to produce all-or-none plateau potentials. Further experimental studies of extrasynaptic glutamate are needed to clarify these issues in different brain regions.

## Data availability statement

The datasets presented in this study can be found in online repositories. The names of the repository/repositories and accession number(s) can be found at: https://github.com/danieltrpevski/Spillover/tree/master and on ModelDB (McDougal et al., 2017).

## Author contributions

DT designed the study, performed the simulations, and wrote the first draft of the manuscript. ZK contributed to the study's design and was involved in the simulations. IC performed the parameter estimation from experimental articles and wrote sections of the manuscript. JHK supervised the project. All authors contributed to the article and approved the submitted version.

## Supplementary material

Video S1 Video animation of the plots in Figures 3_D1_, 3_D2_. The trajectory traversing the phase plot in the upper panel corresponds to the voltage timecourse in the lower panel. For details see figure legend of Figures 3_D1_, 3_D2_.
