# Supplementary material for: Glutamate spillover drives robust all-or-none dendritic plateau potentials—an in silico investigation using models of striatal projection neurons
Source: Front Cell Neurosci. 2023 Jun 29;17:1196182. doi: 10.3389/fncel.2023.1196182 (PMC10352111; doi:10.3389/fncel.2023.1196182)
Supplement: Supplementary file 2 [file Presentation_1.pdf]

Supplementary Material

1 GATING FUNCTION CALCULATION

As shown in Jahr and Stevens (1990), it is possible to express the gating function, $g(V)$, in terms of the transition rates. Specifically:

$$g\left(V\right) = \frac{1}{1+\frac{(a_1+a_2)(a_1B_1+a_2B_2)}{Aa_1(b_1+B_1)+Aa_2(b_2+B_2)}}. \tag{S1}$$

Considering that from the single-channel analysis it follows that b_1 and b_2 are much larger that B_1 and B_2, the following is a good approximation:

$$g\left(V\right) \approx \frac{1}{1+\frac{(a_1+a_2)(a_1B_1+a_2B_2)}{Aa_1b_1+Aa_2b_2}}. \tag{S2}$$

Moreover, when the magnesium concentration ($[Mg^{2+}]$) is greater than a few hundred micromolar, a_2 becomes much larger than a_1 since the former increases linearly with $[Mg^{2+}]$ while the latter is independent of it, and hence $g(V)$ in physiological $[Mg^{2+}]$ can be further approximated as:

$$g\left(V\right) \approx \frac{1}{1+\frac{(\cancel{a_1}+a_2)(\cancel{a_1B_1}+a_2B_2)}{\cancel{Aa_1b_1}+Aa_2b_2}} \approx \frac{1}{1+\frac{\cancel{a_2}B_2a_2}{A\cancel{a_2}b_2}} = \frac{1}{1+\frac{B_2a_2}{Ab_2}}. \tag{S3}$$

Under the assumption that $[Mg^{2+}]$ is greater than a few hundred micromolar, we can approximate the gating function using the transition rates of the 3-state model. In particular, from eq. S2 if we divide by $(a_1 + a_2)$ both numerator and denominator we obtain:

$$g\left(V\right) \approx \frac{1}{1+\frac{(a_1+a_2)(a_1B_1+a_2B_2)/(a_1+a_2)}{A(a_1b_1+a_2b_2)/(a_1+a_2)}}, \tag{S4}$$

and considering that $a = a_1 + a_2$, $B = B_1a_1/(a_1 + a_2) + B_2a_2/(a_1 + a_2)$, $b = b_1a_1/(a_1 + a + 2) + b_2a_2/(a_1 + a_2)$, we can write:

$$g\left(V\right) \approx \frac{1}{1+\frac{aB}{Ab}}, \tag{S5}$$

Finally, taking into account the rate constants (see Table 1), the gating function can be written in its most commonly used form:

$$g\left(V\right) = \frac{1}{1+\eta\,\left[Mg^{2+}\right]e^{-\alpha V}} \tag{S6}$$

Specifically from S3, since:
$a_2 = C \cdot e^{(-0.045 \cdot V - 6.97)}$,
$b_2 = e^{(0.017 \cdot V + 0.96)}$,
$A = e^{-2.847}$,
$B_2 = e^{-3.101}$

we obtain S6:

$$g(V) \approx \frac{1}{1+\frac{e^{-3.101}\cdot C\cdot e^{-0.045\cdot V-6.97}}{e^{-2.847}\cdot e^{0.017\cdot V+0.96}}} = $$
$$= \frac{1}{1+e^{(-3.101-6.97+2.847-0.96)}\cdot C\cdot e^{(-0.045-0.017)\cdot V}} =$$
$$= \frac{1}{1+\eta\,\left[\mathrm{Mg}^{2+}\right]e^{-\alpha V}},$$

where $\eta = e^{(-3.101-6.97+2.847+0.96)} \approx 0.00028$ and $\alpha = 0.045 + 0.017 = 0.062$.

2 SUPPLEMENTARY FIGURES

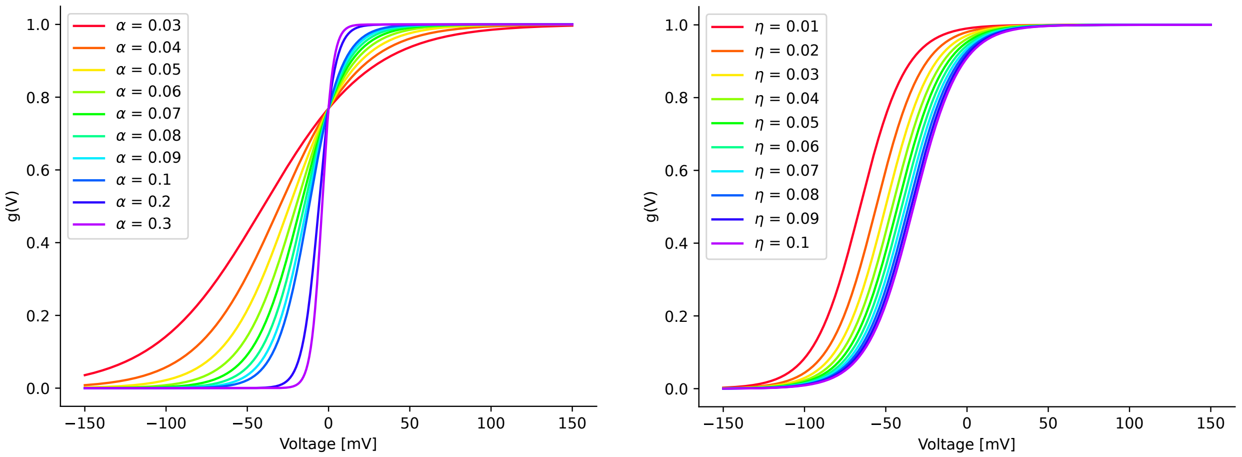

Figure S1: Effects of the parameters α and η on the shape of the gating function. Left: Increasing values of α increase the steepness (slope) of the sigmoid curve. Right: Increasing values of η shift the gating function to higher voltages. Linear increases in η do not correspond to linear shifts along the voltage axis. In fact, the product $\eta[\mathrm{Mg}^{2+}]$ determines the shift along the x-axis, and the exact value of $V_{1/2}$, the voltage value where half of the receptors are blocked by Mg^{2+} is given by $V_{1/2} = \frac{\ln \eta[\mathrm{Mg}^{2+}]}{\alpha}$.

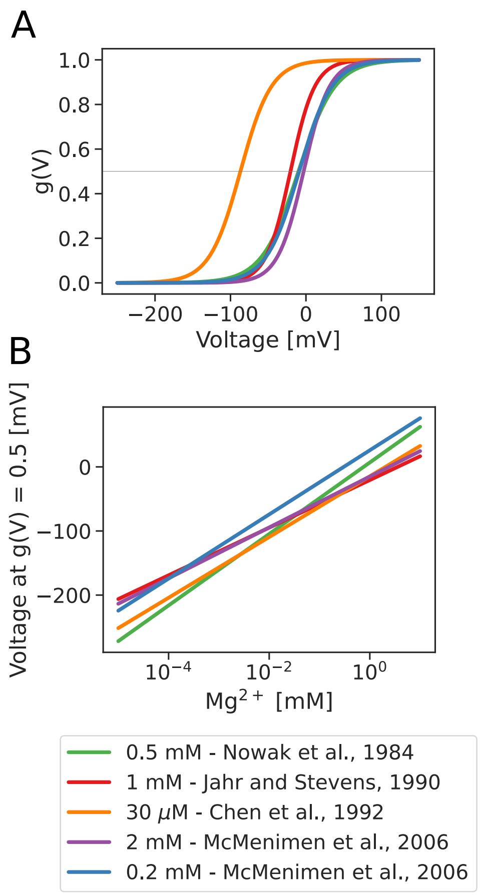

Figure S2: (A) Sigmoid curves for the parameter values in Table 2 of the main text. Grey horizontal line indicates $g(V) = 0.5$. (B) Relationship between the voltage at which the conductance is half maximal and Mg^{2+} concentration. Lines represent the equation $V([\mathrm{Mg}^{2+}]) = \frac{1}{\alpha}\ln\left(\frac{1}{\eta[\mathrm{Mg}^{2+}]}\right)$, hence lines with smaller slopes indicate higher values of α, i.e. steeper gating functions, and larger vertical shifts indicate smaller values of η. Note that the plot is in semi-log scale.

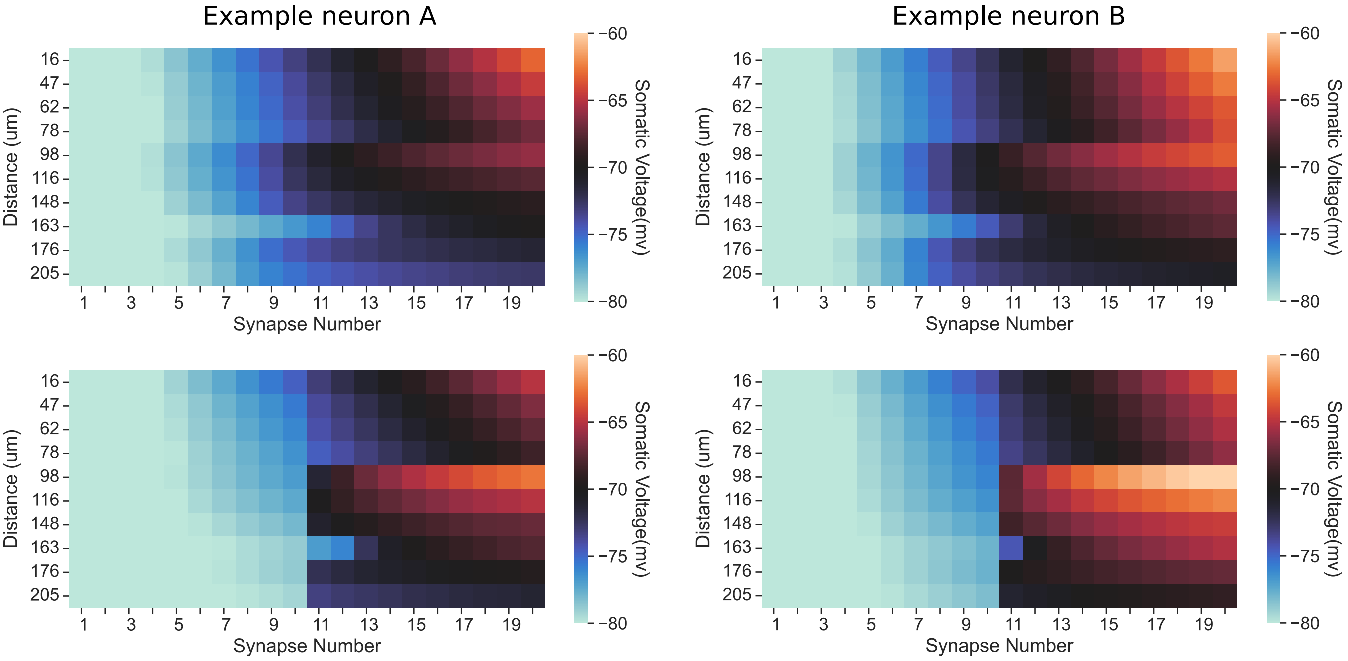

Figure S3: Distance dependence of the somatic amplitude of a plateau potential. Plateaus were evoked in 10 different dendrites, at the indicated distance from the soma. Amplitudes of somatic voltage are averages over 20 trials. We have used two SPN models from the library in Lindroos and Hellgren Kotaleski (2021) which have different excitability (cell A less excitable than cell B). The peak deplorarizations evoked with clusters close to the soma are not due to plateau potentials, but to fast glutamate-evoked responses. The relative increase in amplitude for more distal locations (here clearly visible at 98 μm) correspond to plateau potentials. The somatic amplitude of the plateau potentials decreases with more distally placed synaptic clusters, as expected. Note the different heatmap scale from the plots in the main text.

REFERENCES

Jahr, C. and Stevens, C. (1990). Voltage dependence of NMDA-activated macroscopic conductances predicted by single-channel kinetics. The Journal of Neuroscience 10, 3178–3182. doi:10.1523/jneurosci.10-09-03178.1990

Lindroos, R. and Hellgren Kotaleski, J. (2021). Predicting complex spikes in striatal projection neurons of the direct pathway following neuromodulation by acetylcholine and dopamine. Eur J Neurosci 53, 2117–2134
